# Exploring non-physician care professionals’ roles in cancer multidisciplinary team meetings: A qualitative study

**DOI:** 10.1371/journal.pone.0263611

**Published:** 2022-02-03

**Authors:** Melissa Horlait, Melissa De Regge, Saskia Baes, Kristof Eeckloo, Mark Leys

**Affiliations:** 1 Faculty of Medicine and Pharmacy, Department of Public Health, Vrije Universiteit Brussel, Brussels, Belgium; 2 Faculty of Economics and Business Administration, Department of Marketing, Innovation and Organisation, Ghent University, Ghent, Belgium; 3 Strategic Policy Cell, Ghent University Hospital, Ghent, Belgium; 4 Faculty of Medicine and Health Sciences, Department of Public Health and Primary Care, Ghent University, Ghent, Belgium; Universiteit van Amsterdam, NETHERLANDS

## Abstract

The growing complexity of cancer care necessitates collaboration among different professionals. This interprofessional collaboration improves cancer care delivery and outcomes. Treatment decision-making within the context of a multidisciplinaire team meeting (MDTMs) may be seen as a particular form of interprofessional collaboration. Various studies on cancer MDTMs highlight a pattern of suboptimal information sharing between attendants. To overcome the lack of non-medical, patient-based information, it might be recommended that non-physician care professionals play a key patient advocacy role within cancer MDTMs. This study aims to explore non-physician care professionals’ current and aspired role within cancer MDTMs. Additionally, the perceived hindering factors for these non-physician care professionals to fulfil their specific role are identified. The analysis focuses on nurses, specialist nurses, head nurses, psychologists, social workers, a head of social workers and data managers. The results show that non-physician care professionals play a limited role during case discussions in MDTMs. Neither do they actively participate in the decision-making process. Barriers perceived by non-physician care professionals are classified on two main levels: 1) team-related barriers (factors internally related to the team) and 2) external barriers (factors related to healthcare management and policy). A group of non-physician care professionals also belief that their information does not add value in the decision-making proces and as such, they underestimate their own role in MDTMs. To conclude, a change of culture is needed towards an interdisciplinary collaboration in which knowledge and expertise of different professions are equally assimilated into an integrated perspective to guarantee a true patient-centred approach for cancer MDTMs.

## Introduction

The growing complexity of cancer care necessitates collaboration among different professionals as evidence suggests that the collaboration between professionals improves cancer care delivery and outcomes [1]. According to the World Health Organization (WHO), interprofessional collaboration is defined as *“two or more individuals from different backgrounds with complementary skills interacting to create a shared understanding that none had previously possessed or could have come to on their own”* [2, p36]. Interprofessional collaboration in cancer care is typically carried out by means of multidisciplinary team meetings (MDTMs). These meetings consist of multiple professionals involved in the delivery of care for cancer patients uniting weekly or fortnightly to review patient cases and discuss treatment options with the aim of agreeing upon an individual treatment recommendation for each cancer patient [3].

MDTMs are proven to be essential to cancer care as they improve decision-making, coordination and communication [4]. Above that, health professionals report strong benefits from MDTM related to support for further patient management and professional competence development [5].

However, previous research also illustrate that mainly medical information is exchanged during the MDTMs [4]. Alongside this medical information patient-related factors should be taken into account to ensure true holistic treatment decision-making [3,4]. Patient-related factors include more general information related to health and performance status, comorbidity, risk factors, psychological factors, vulnerabilities and aspects such as supportive network, psychosocial needs and patients’ treatment preferences. Evidence shows that consideration of these patient-related factors lead to more robust treatment decisions with a higher likelihood of implementation of the treatment plan [6–9].

Ideally, this could be reached by shared decision-making with the patients themselves. Shared decision-making involves informing the patient about the diagnosis, the different treatment options, eliciting their goals and collaborating on equal footing when deciding on treatments [10]. However, due to practical reasons, patients generally do not attend MDTMs [11–13]. That of course makes it especially challenging to incorporate individual patients’ perspectives and preferences [4].

And indeed, various studies on cancer MDTMs highlight that patient-related information and patient preferences are generally considered to a lesser extent. Therefore, treatment decisions made in MDTMs are primarily made on the basis of medical information at the expense of an individualized, patient-centred treatment decision [3,14–17]. Evidence shows that insufficient consideration of non-medical, patient-based information [15,18–21] may eventually lead to a need for further conversations with the patient and repeated case presentation in an MDTM, which delays treatment [6,17,22,23].

To overcome this lack of non-medical, patient-based information, it might be recommended that designated team members act as patients’ advocates during the team meetings [19]. Literature refers repeatedly to (specialist) nurses or other non-physician care professionals such as psychologists who can adopt this role [24–26]. Besides the clinical skills which are unique to their discipline and their own clinical wisdom and knowledge that might enhance a true holistic approach for the MDTMs, they are also more likely to hold particular patient centred information. Therefore, they may be well placed to advocate patients’ unique circumstances or concerns during discussion [20,27].

Treatment decision-making is, however, still considered to be a solely medical task [28]. Medical professionals themselves define the particular aim of an MDTM as purely clinical, i.e. as intended to review and discuss patient cases from different medical perspectives to advise appropriate treatment grounded in evidence-based treatment guidelines [28]. Studies have shown that nursing staaf who may be able to contribute information on psychosocial matters generally participate far less frequent [22,29,30]. Furthermore, when nurses do attend or even speak up during the meetings, their contributions can be overlooked or ignored by other members of the team [17,22].

Very few studies have investigated the specific role of non-physician care professionals in treatment decision-making [31]. Assuming that non-physician care professionals can play a key patient advocacy role and that this may be a meaningful step forward towards a true multidisciplinary and patient-centred practice within cancer MDTMs, this study aims to explore non-physician care professionals’ perceived current and aspired role within cancer MDTMs. Additionally, it sought to identify the perceived hindering factors for these non-physician care professionals to fulfil their specific role.

## Methods

To ensure methodological rigour, we follow the COREQ checklist specifically developed to promote explicit and comprehensive reporting of qualitative studies using interviews and focus groups as methods for data collection [32].

### Study design

This study adopted an exploratory qualitative design using focus groups and in-depth interviews.

### Participants and recruitment

Participants were recruited with the following inclusion criteria: (1) non-physician cancer care professionals who (2) had worked for at least one year in a medical oncology department of a Flemish institution (academic or general hospital). Efforts were made to recruit participants with varied age, work experience, gender, and discipline through contacting academic and non-academic medical oncology departments. Eligible nurses, psychologists and social workers showing interest were invited for an individual in-depth interview. In a second phase, additional participants for the focus groups were attracted via snowball sampling among professional contacts of both the participants and the researchers.

### Study setting

In Belgium, the Multidisciplinary Oncology Consult (MOC) exists as a specific, legally regulated and mandatory type of MDTM. It includes a financial incentive for participating physicians paid by the National Institute for Health and Disability Insurance [33]. The MOC is legally described as a single consultation per individual patient but because of organizational convenience these consultations are clustered in a collective meeting for all patients (generally per tumour group) and are named as the ‘MOC meetings’[3]. The purpose of the MOC is to discuss the patient case and to develop a treatment plan and follow-up for every individual patient [33,34]. The course of the MOC meetings generally consists of two phases: a first phase of case discussion with information exchange between the different participants of the meeting, followed by a phase of decision-making [3].

The Belgian law stipulates four specific situations in which the discussion of a patient case in a MOC is mandatory: (1) when an oncological treatment deviates from the hospital’s oncology manual, (2) when re-irradiation of the same target zone is envisaged within 12 months of the start of the first radiotherapy, (3) when chemotherapy is delivered with a drug that is in its first reimbursement phase, and is to be monitored by experts as such, and (4) from 2007 onwards, for every new breast cancer diagnosis treated in a formally recognized breast clinic [33]. Also, according to the legislation, a MOC must be chaired by a (medical) MOC coordinator (preferably with specific cancer expertise) and must count at least four different medical specialists (for example, specialists in oncology, radiotherapy, surgery, internal organs, or pathology) who belong to the hospital staff, as well as one extramural participant (e.g. the general practitioner). The non-physician oncology staff members (i.e. psychologists, nurses, social workers and data managers who register data for the national cancer centre) are not legally bound to participate in the MOC. As a result, it has been observed that their attendance varies considerably between hospitals and departments [3,34].

### Data collection

Data were collected between July 2019 and January 2020, by the main researchers (SB) and (MH) both certified clinical psychologists working in an academic context as researchers. Interviews were conducted to explore individual opinions and perspectives among nurses, psychologists and social workers concerning their role in MOC meetings. Focus groups were organized to understand shared experiences regarding their participation at MOC meetings and to uncover the reason behind individuals’ actions, beliefs, perceptions and attitudes [35].

A semi‐structured topic guide, which had been pre-discussed within the research team, was used in the interviews and focus groups. The topic list addressed the following themes: 1) the perceived current role of non-physician care professionals in the MDTMs 2) the perceived hindering factors in fulfilling an active role in MDTMs and 3) the aspired role of non-physician care professionals in the MDTMs. Work-related background and some socio-demographic data were collected via a brief pre-interview questionnaire. During the individual interviews, only the main researcher (SB) and the participant were present at a location chosen by the participant according to his/her preferences. The focus groups were performed in appropriate meeting rooms in two different hospitals. They were conducted by a moderator (SB) and an assistant moderator (MH). The moderator led the discussion and attempted to involve all participants in an open conversation. The assistant moderator mainly observed, but was able to participate orally if needed. All individual interviews and focus groups were audio-recorded and transcribed verbatim.

### Data analysis

Transcripts were analysed according to the principles of thematic analysis [36], using Nvivo V12 for data management. A thematic analysis was chosen as it allows for a theoretically flexible approach, while also being a solid tool to analyze data in a rich and detailed way. Thematic analysis is generally carried out in six phases: data familiarisation, initial coding, searching for higher-level themes, reviewing themes, defining and naming themes and finally compiling a report of the analysis [36].

First, authors MH, SB and ML read the transcripts independently to obtain a first impression of the main topics emerging from the data. SB subsequently proceeded independently with identifying and labelling meaning units (i.e., fragments of text containing information about the aim of the study) with a code. Related codes were sorted and placed into code groups, corresponding to the preliminary themes. The preliminary themes were then discussed within the research team allowing refinement of adjustment of the themes. Once agreement on the themes was reached, they were further divided into sub-themes. Finally, conclusions of the analysis were drawn during periodic discussions within the research team.

### Ethical considerations

This study is part of a larger research project on multidisciplinary teams and team based communication in oncology, for which ethical approval was granted by the central Medical Ethics Commission of the Brussels University Hospital (BUN: 143201939232). All participants received written and oral information regarding the study and written informed consent was obtained before interviews began. Confidentiality was assured for all participants and data from this study was anonymized and stripped of personally identifying elements.

## Results

A total of 32 non-physician cancer care professionals were included in the study; 28 were females and four males. The group involved 20 nurses (of which two were specialist nurses and five head nurses), seven psychologists, three social workers (with one head social worker) and two data managers. Fourteen participants were interviewed individually, and two duo-interviews were conducted. The two focus groups each counted seven participants (see Table 1).

**Table 1:** Different non-physician care professions included per type of data collection.

Individual interviews	Duo interviews	Focus group 1	Focus group 2
3 psychologists 7 oncology nurses 1 specialist nurse 1 head nurse 2 social workers	2 oncology nurses 2 data managers	2 psychologists 1 specialist nurse 1 head social worker 3 head nurses	2 psychologists 4 oncology nurses 1 head nurse

Three participants had less than five years of experience in cancer care, sixteen participants had between five and fifteen years of experience, while thirteen had over fifteen years of experience. Two psychologists and five head nurses reported not attending MDTMs on a regular basis but rather sporadically. Twenty-three participants were working in a general (non-academic) hospital, while the other nine were working in an academic cancer centre. All worked in one of the following tumour-specific departments: breast cancer, cervix cancer, head and neck cancer, pneumology, haematology, digestive oncology, neurologic oncology (see Table 2).

**Table 2:** Overview of MOC attendance per profession by gender, years of experience and type of hospital.

	Gender	Experience	Hospital
Nurses	17 female 3 male	8 <15years 12 >15years	15 non-academic 5 academic
Psychologists	7 female	5 <15years 2 >15years	6 non-academic 1 academic
Social worker	2 female 1 male	3 <15years	2 non-academic 1 academic
Data managers	2 female	2 <15years	2 academic

The results of the interviews and focus groups are presented below, following the three main study aims: 1) to identify the current role perception of non-physician care professionals during MDTMs, 2) to identify the perceived hindering factors in fulfilling this role in MDTMs and 3) to explore the aspired role of non-physician care professionals in MDTMs.

### How do non-physician cancer care professionals perceive their current role in the MDTM?

#### Passive role perception

The entire group of social workers, the (mainly) less experienced nurses and the majority of psychologists, perceive their role during MDTMs as passive. In their view, the MDTM is a forum in which medical professions, but not other professionals, collectively decide upon the best possible treatment and care plan for each cancer patient. They do not see themselves as holding a significant role and therefore do not actively participate in the case discussions and decision-making process. However, they argue that being present at a moment where a lot of information regarding the patient is bundled and shared enables better adjustment of care and support to the specific situation of the patients. As a result, this group of non-physician care professionals only attend the meetings with the aim of gathering as much information as possible about the patient and on the treatment decision made within the MDTM.

Nurses and social workers in particular report that it is essential for them to be fully informed about the patients’ diagnosis and co-morbidities, medical history and current disease course. For nurses, this information is important to pursue the appropriate care pathway for the individual patient. Social workers consider this information as a necessity to make follow-up arrangements (e.g. home care services, etc.). For both professions, having this kind of information might also enhance effective, patient-centred communication in their encounters with the patients.

***“I attend MOCs to know what treatments patients undergo*. *If they are going to have surgery*, *I know that I need to visit that patient post-surgery to see if he/she needs home nursing or insurance information or anything*.*”*** (P2, social worker)

The majority of the interviewed psychologists are likely to regard themselves as outcasts of the core care team. The need for psychosocial or psychotherapeutic interventions for a cancer patient is multifactorial in nature, involving the complexity of the cancer diagnosis, the individual psychosocial situation of the patient and the possible (physical or psychological) consequences of the treatment trajectory. Having reviewed the patient’s status, the psychologist tries to identify objectives of psychological support to further determine which of many counselling or psychotherapeutic methods are best suited for the patient. However, the psychologists interviewed in this study report that this assessment process is mostly initiated later in the patient trajectory. Furthermore, it is conducted as a separate task, specific to their profession, which they perform independently of the initial treatment decision-making process. Moreover, psychological counselling or support does not necessarily have to be initiated for every cancer patient discussed within a MDTM. Although they consider that the process of treatment decision-making might affect the patients’ illness perception and acceptance, there is no added value for psychologists to be present at the very moment of multidisciplinary treatment decision-making. As one psychologist explains, ***“Mainly*, *the MOC is about being informed on medical perspectives and treatment possibilities [*…*]; we also gain more insight into the level of difficulty in the diagnostic process*.*"*** (P7, psychologist).

In addition, some non-physician care professionals are given the task of completing the mandatory cancer registration. Most of the data needed for registration is presented during case discussions in MDTM. In academic or large non-academic hospitals, specific data managers perform this task. In smaller hospitals, a nurse is often responsible for this task.

One nurse explains: ***“I am present at the MOC to collect information of my patients*, *but secondly I have to enter cancer registry"*** (P16, nurse).

#### Active role perception

Those who perceive their role to be actively involved in the MDTMs are mainly the specialist nurses, experienced nurses and a small proportion of psychologists. They participate in the case discussions by sharing psychosocial or other patient-related information, believing that this might provide a more holistic perspective for what is generally mere medical information. Specialist nurses consider themselves as the ‘coordinators of care in oncology’; they have multiple contacts with patients, monitor the patients’ trajectory and are updated with most psychosocial aspects of the patients’ cases. The specialist nurses accentuate, more than other nurses, the importance of communicating patient related information (physical or psychosocial) to team members in MDTMs. This role in MDTMs is also perceived by experienced nurses who work in various tumour-groups. Experienced nurses, more than other nurses, emphasise that they have confidence in their expertise, feel part of the team and perceive their information as being of added value in case discussions.

***“Everyone may speak up [*…*] that is what you see growing in all those years*. *In the beginning*, *you are a lonely person on your island; you have to free your path*. *After a while*, *they know how important your information is*.*”*** (P30, nurse)

Only a few psychologists, mainly working in non-academic hospitals, consider themselves as potential advocates for patients in MDTMs. If they have been counselling the patient (and relatives) for some time, they know about the patient’s values, life goals and preferences. In that case, it might occur that they attend the MDTM (sometimes upon the request of patients themselves). As one psychologist states, ***“During MOCs*, *where the patient is absent*, *we are his advocate and need to speak up his/her words”*** (P32, psychologist). Remarkably, they do not actively participate in the decision-making processes.

Data managers who complete mandatory cancer data registration do not participate in case discussions; however, when a patient is discussed in more than one MDTM (e.g. meeting in another tumour-related department or a follow-up meeting), data managers pass patient-related information from one MDTM to another as requested by physicians.

***“We pass information from one MOC to another*, *when the patient is discussed in two different MOCs"*** (P13, data manager).

### What are the barriers for non-physician cancer care professionals taking an active role during MDTMs?

Barriers perceived by non-physician care professionals are classified on two main levels: 1) team-related barriers (factors internally related to the team) (see Table 3) and 2) external barriers (factors related to healthcare management and policy) (see Table 4). Within each of these main levels, barriers are further divided in subcategories.

**Table 3:** Team-related barriers to non-physician cancer care professionals taking up an active role during MDTMs.

Type	Subcategory	Mechanism	Professions affected
**Related to the organization of the team meetings**	Team composition	A large varied group of medical professionals in the meeting hinders active exchange of information.	Less experienced nurses and social workers
	Spatial arrangements	Non-physician professionals are seated on the peripheries hindering participation in case discussions.	Social workers and a group of nurses
	Agreements regarding division of roles	Lack of (informal) agreements leads to an unclear role and ambiguous expectations among physicians.	Social workers and a group of nurses
	Time management	Time pressure hinders non-physician professionals to exchange patient-cantered information.	Psychologists, nurses and social workers
**Interaction-related**	Team climate	A medical focus prevails in MDTMs, which hinders active participation by non- physician professionals. This is strengthened by legal regulations but can be mediated by hospital culture. The attitude of the coordinator of the MDTM is a key factor.	The majority of psychologists, nurses and social workers
**Personal**	Professional beliefs	Non-physician professionals’ belief that treatment decisions are based on solely medical information, and that patient-related information has no added value in the decision-making process, hinders active case participation.	The majority of psychologists and nurses
	Personal skills	Lack of self-confidence and assertiveness hinders active case participation.	Psychologists, nurses and social workers
	Work experience	Lack of experience in oncology as well as the lack of experience in the MDTM hinders active case participation. This can be mediated by personal skills.	Psychologists, nurses and social workers

**Table 4:** External barriers to non-physician cancer care professionals taking up an active role during MDTMS.

Type	Subcategory	Mechanism	Professions affected
**Structural**	Timing of the meeting in patients’ trajectory	Since MDTMs are organized at the beginning of a patient’s trajectory, a group of non-physician professionals lacks information on the patient, which hinders active case participation.	The entire group of psychologists, nurses and social workers working in smaller (non-academic) hospitals
	Legal regulations	Legal regulations favour medical professions, which strengthens the medical focus in MDTMs.	Psychologists, nurses and social workers
	Standardization	Standardization of treatment and care leads, in a large number of cases, to standard medical decisions being made where no input of non-physician professionals is needed.	Psychologists, nurses and social workers
**Process**	Hospital culture	Lack of patient-centred approach of the hospital strengthens the medical focus in MDTMs, hindering active participation of non-physician staff	Nurses

#### Team-related barriers

*Barriers related to the organization of team meetings***. Composition of the team.** Less experienced nurses and social workers working in non-academic hospitals generally stress that the size and composition of the team during MDTM impacts the process of information exchange. A large group of participants, coming from a variety of medical disciplines, deters the non-physician care professionals from speaking up. It also fosters the concern that non-medical information may not be relevant for the attending physicians—some of whom will never even meet the patient in person (e.g. pathologist, radiologist, physician from nuclear medicine, etc.). A specialist nurse explains: ***“You have the feeling that when you are sitting at the table with many physicians*, *cases need to be discussed fast and there is less room to share those psychosocial aspects"*** (P3, specialist nurse)

This reluctance can, however, be mediated with the presence of a general practitioner. The patient’s specific situation, support resources, values and preferences are more likely to be discussed when the general practitioner is participating in the meetings.

***“You notice that when a general practitioner is present*, *the social context is more present in discussions*.*”*** (P10, nurse)

#### Spatial arrangements

In almost every meeting, physicians are seated centrally while non-physician care professionals join them in the peripheral space. Case discussions are hard to follow when seated peripherally, which makes it even more difficult to actively participate in the discussions.

***“I do not know if they would hear me if I would say something*. *They [the physicians] are sitting in front and it is very difficult to understand them*. *You have to prick up your ears to be able to understand what they are saying*.*”*** (P2, social worker)

#### Role division

In most hospitals, there is a lack of (informal) agreements regarding non-physician care professionals’ role. This breeds ambiguous expectations of physicians towards social workers and nursing staff. Social workers feel particularly unrecognized and undervalued in their role and in fulfilling tasks in a cancer patient’s trajectory. In most hospitals, psychologists are not even invited to attend the MDTMs.

***“There is never an agreement on what I am allowed*, *or should*, *do or not do; on what I should expect and what the physicians expect from me [*…*] I have only said something two or three times and I felt like a burglar*.*”*** (P15, social worker)

#### Time management

The high workload in MDTMs leads to significant time pressure. Time pressure has been reported by all three groups of non-physician care professionals, mainly active in academic hospitals, as a barrier for patient-cantered information exchange during team meetings. One nurse explains, ***“It goes very quickly [*…*] no attention is given to the patient themself or to their psychosocial aspects*. *Medical discussions happen very fast*, *to be honest*.*"*** (P16, nurse).

#### Interaction related barriers

*Team climate*. The majority of psychologists, nurses and social workers perceive a solely medical focus driving MDTMs. The majority of social workers and a small group of nurses experience disinterest of physicians towards psychosocial information. Moreover, even when non-medical information about the patient is being shared, this kind of information is often not taken into account during the decision-making process. One nurse opines that ***“Maybe they [physicians] will listen out of courtesy*, *but nothing constructive will happen of it anyway*. *Therefore*, *I do not want to put effort and energy into it*.*”*** (P16, nurse).

The attitude of the coordinator of the meeting towards non-physician involvement seems to be key in either reinforcing or reducing a medical focus. Speaking of non-medical information being shared, one nurse says, **“*The coordinator of the MOC does not tolerate it*, *in my opinion*. *It has to go quickly because there are a lot of cases*. *I can imagine…well [*…*] regrettable*"** (P17, nurse).

#### Personal barriers

*Professional beliefs*. Psychologists seem to have a strong belief that treatment decisions should be solely based on medical information and see no added value for patient-related information in the decision-making process. This belief hinders their likelihood to actively participate or attend the MDTMs. This viewpoint is also shared by a group of head nurses and social workers, which may have an inhibiting effect on nurses and social workers in their team.

**“*Then this [psychosocial information] is shared*. *But does it have any impact*? *No*. *Because as a physician you have to say*: *from a medical point of view*, *this is the best solution [*…*]*. *Will his or her treatment be different*? *I do not think so*. *So*, *is it relevant for the MOC*? *I do not think so…”*** (P1 psychologist)

#### Personal skills

Self-confidence and assertiveness are perceived by almost all respondents of the three professions (psychologists, nurses and social workers) as necessary to speak up during MDTMs. The lack of these personal skills hinders participation in case discussions. One psychologist asserts, ***“What I have to share is important*. *It is another aspect*, *but it is also important*. *You need to have that self-confidence”*** (P26, psychologist).

#### Work experience

Respondents from all three professions, stress that the lack of experience in oncology specifically as well as the lack of experience in MDTMs working hinders active participation. This can be mediated by personal skills (see supra).

**“*The longer I work in oncology*, *the easier the communication gets*, *because you have a better relationship with the physicians*. *And so the physicians know that I am capable of distinguishing between relevant and non-relevant information”*** (P3, specialist nurse).

#### External barriers

*Structural barriers***. Timing of the meeting in patient’s trajectory.** The discussion of a patient’s case within a MDTM is generally planned at the beginning of a patient’s trajectory, mostly immediately after cancer diagnosis. As a consequence, chances are higher that some of the non-physician care professionals (e.g. the psychologist) have not yet met the patient. Furthermore, in most hospitals, the support of a psychologist is not a standard procedure for every cancer patient. Nurses and social workers, on the other hand, are the first in line to meet patients. However, in some (smaller) hospitals, nurses and social workers work for more than one tumour-group and do not have enough time to meet all patients in preparation for the MDTM. In this case, they have little or no information to share during the discussion. One psychologist explains, “***I do not know*, *or may never meet*, *a large proportion of the patients discussed within the MOC”*** (P7, psychologist).

#### Legal regulations

Almost all respondents stress that legal regulations favour the physician professions, which strengthens the medical focus in MDTMs and discourages a true multidisciplinary and collaborative team approach.

**“*In the MOC system*, *only physicians are funded*. *There have to be a minimum of three or four physicians to actually receive money […] that is wrong*, *because that says something about the quantity but nothing about quality”*** (P29, nurse).

#### Standardization

Some, if not most, of the patient cases being discussed during a MDTM can be decided upon following standardized, evidence-based treatment guidelines. The decision-making process for these cases might requires a more brief discussion, which means that input from all those present is not needed at that moment.

**“*Certain diagnoses have a standard treatment anyways … The physician indicates this standard treatment to the patient*. *Afterwards*, *a MOC is organized*, *but the treatment plan has already been made*. *Then it [the MOC] is purely an administrative matter*.*”*** (P10, nurse)

#### Process barriers

*Hospital culture*. The lack of a patient-centred hospital culture strengthens the medical focus. On the contrary, the medical focus can be mediated by a patient-centred hospital culture. One nurse elaborates, **“*How you install legal regulations in the hospital*, *and whether you have a clear vision around it (patient-centred care) in your hospital*, *also has an impact*.*"*** (P29, nurse).

### What kind of role do non-physician cancer care professionals aspire to in treatment decision-making?

Mutual agreements on the role division between physicians and nurses, or within the team, seem to be key in enabling an active role for non-physician care professions in MDTMs. However, role descriptions may differ between hospitals as well as between tumour-related departments due to a lack of legal regulations or formal guidelines.

Nevertheless, the majority of non-physician care professions do not see a dedicated active role for themselves in the treatment decision-making process. They do, however, feel that their current role can be optimized. They suggest the use of a tool to facilitate their input (e.g. checklist). The group of nurses, in particular, aspires for a more open attitude among physicians towards their information input.

***“I think physicians’ attitudes in the MOC have to be questioned*. *When the MOC coordinator is informed about all the attendees from various professions*, *but does not give [everyone] an opportunity to speak [*…*] the attitude must change*, *the agreements within the MOC have to change*.”** (P17, nurse)

Specialist nurses and experienced nurses are satisfied with their current active role. They describe themselves as having ‘grown into it’ and experience no restraint in actively participating in case discussions. This group identifies improvement in terms of interdisciplinary decision-making. Ideally, communication and integration of information between medical and non-physician care professionals should also be extended in daily practice (before and after meetings) to foster shared decision-making. A small group of psychologists shares this opinion. A head nurse affirms: ***“I actually think there should be more focus on interdisciplinary training”*** (P31, head nurse).

For the majority of the psychologists the ‘gathering information-role’ is sufficient since the referring physician already addresses psychosocial information when needed (e.g. strong patient preferences or specific concerns). Therefore, the majority of psychologists remain critical of their time spent during the meetings. One psychologist comments: ***“Sometimes*, *I question those meetings… what a waste of time”*** (P1, psychologist).

## Discussion

This study explored non-physician cancer care professionals’ current and aspired role in MDTMs. The MOC has been, since 2003, a legally regulated form of MDTM at Belgian oncology departments. Some of the legal criteria surrounding the MOC address details about the attendance of medical professionals; for example, the MOC is reimbursed when at least four (defined) medical disciplines are represented [3]. Although this benefits patients’ quality of care, attendance of oncological nurses and other non-physician care professionals (such as psychologists and social workers) during the MOC is not regulated by law, making their role in MDTMs rather ambiguous. The current study identifies hindering factors for these non-physician care professionals to fulfil an active role in the MOC meetings. In particular, our analysis focuses on nurses, specialist nurses, head nurses, psychologists, social workers, a head of social workers and data managers.

This study investigates non-physician care professionals’ experience of their current role in MDTMs in oncology. Two groups are distinguished: 1) one group, composed of the entire group of social workers, the majority of nurses and the majority of psychologists, perceive their role in MDTMs as rather passive and limited to information gathering (such as on medical history, current disease course, etc.); 2) another group, composed mainly of experienced nurses and specialist nurses, perceive their role in MDTMs as active, as for the exchange of patients’ non-medical information in MDTMs. However, despite the alternative views of the second group, overall our results show that non-physician care professionals play a limited role during case discussions in MDTMs, a finding that is in line with recent literature on a nurse’s role in multidisciplinary team meetings [26,36–39]. Neither do they actively participate in the decision-making process. This finding is also consistent with previous findings in literature [3,15,17,22,40].

The several hindering factors for non-physician care professionals to take up an active role in MDTMs identified in this study are classified into team barriers (organization of team meetings, interaction dynamics and personal factors) and external barriers (structural and process factors). A new insight into the barriers to non-physician care professionals having an active role in collaborative decision-making are system-related and organizational factors. Although the Belgian government encourages patient-centred care and requires the inclusion of psychosocial information in treatment plans, the legal provisions for MDTMs only make the presence of medical professionals compulsory [3]. Consequently, the process of treatment decision-making is often a purely medical matter. Also previous studies highlight how the dominance of a biomedical perspective during MDTMs have a negative effect on the participation of non-physician care professionals [15,40,41]. Besides basic information on patients’ age and general state of well-being (‘he/she is well’), psychosocial information is rarely shared during decision-making discussions and especially not by physicians [4]. Our study reveals that a group of non-physician care professionals themselves also belief that their information does not have an added value in decision-making and as such, they underrate their own role in MDTMs. This belief is mainly held by psychologists who are likely, as a result, to regard themselves as outcasts of the core care team. Further research is needed to investigate the specific role of psychologists in MDTMs since there are indications that the role of psychologists in meetings might differ from that of nurses.

Research shows that non-physician team members have particular clinical skills which are unique to their discipline and their own clinical wisdom and knowledge which should be incorporated anyhow to enhance a true holistic approach within the MDTMs. What is more, when the patient is known by one or more team members in the MDTM (regardless of their function) patient preferences are included to a greater extent in the decision making process [40], thus affirming the importance.

Another aspect that cannot be ignored is the time associated with taking part in an MDTM. Conducting MDTMs requires significant time investment [42]. Besides being present at the meetings, additional time is also required to prepare the patient case for discussion during the MDTM [43]. With a growing number of case discussions and increasingly complex diagnosis and treatment options, questions regarding the resource-effectiveness of the MDTMs are raised and a risk of decision-making fatigue exists. A possible solution might be to prioritize or streamline case discussions according to case complexity [44,45]. The participants of this study indeed report that not every patient case needs a thorough case discussion (including discussion of the psycho-social aspects).

Further, organizational factors may have an inhibiting effect on the exchange of psychosocial information in collaborative decision-making. The timing of the MDTMs is misaligned with the timing of non-physicians care professionals’ meetings with patients during their trajectory. All three professions explained the difficulty of having a session with the patient to gather their information before MDTMs. Kidger et al. (2009) suggest that patient-related information needs to be considered as early as possible and ideally at multiple points along the treatment journey and that nurses need to take a more central role in discussions on patients [17]. Finally, our results are in line with recent findings of teamwork in acute care. Barriers that hindered teamwork, expressed by nurses working in acute care, include different perceptions of teamwork and the dominance of medical power influencing the interaction dynamics in teams [46]. Another barrier that was commonly experienced relates to the different levels of skill-acquisition in order to function as a team member (i.e. the level of assertiveness and confidence). However, we also highlight the evolving role of the nurse specialist as coordinator between multiple care providers in the oncology patient’s care process [47]. It is therefore likely that the nurse discusses specific (non-medical) problems beforehand with, among others, the attending physician, who than takes this information into account at the MTDMs. Likewise, previous research underlines the importance of an optimal nurse-physician relationship to enhance oncology practice settings [48].

Various strategies are suggested in literature for qualitative multidisciplinary case discussions, such as a solid structure in meetings to ensure that every professional present can contribute in discussions [17], the use of a checklist to ensure all information is included in a decision-making process [14,17,49] and a more central role for nurses in MDTMs (e.g. preparations or chair) [17]. There is also valuable research on streamlining cases [50] to distinguish between complex and standard cases, in which non-physician care professionals mainly play a role in complex cases in providing a holistic profile of the patient [50].

Based on our study, we recommend MTDMs having clear objectives; since the MDTM is experienced as a medical forum, agreements should be made where psychosocial and other information should be included in the decision-making process. As MDTMs are legally regulated, a description of the role to be played by non-physician care professional should also be foreseen. In addition to these strategies, a change of culture is needed in cancer care towards a true interdisciplinary collaboration. Interdisciplinary collaboration is inherent to patient-centred care and required in cancer care. In interdisciplinary collaboration, the knowledge and expertise of different professions are equally assimilated into an integrated perspective [51]. It is clear that non-physician members have valuable and essential contributions because of their unique disciplinary skills but a true integration of these professionals and a proper mechanism to contribute their information to the decision-making process is essential to guarantee qualitative decision-making by including all aspects and preferences of the individual patient.

As such, efforts should be made to broaden this currently perceived medical forum and to enhance patient-related information sharing. Above that, it may well be that outside the context of the MOC, which are designed to decide on the treatment plan, there are more patient-centred interprofessional meetings in which participation of all disciplines (e.g. MDTM for patients with acquired brain injury), and where all respective disciplines are heeded [52,53]. It might be interesting to investigate how this can be organised and how these differ from a MOC. Likewise, to empower the non-physician care professional they could be trained in improving their leadership- and mentorskills especifically for interprofessional meetings, as should the chair be trained to specifically ask for interprofessional contributions.

In the same way it is too bluntly to state that physicians are not willing to take up a patient advocacy role. This study shows that in MDTMs physicians predominantly contributes biomedical information and not information about patients’ psychosocial circumstances even though they are likely to know this. We may assume that it is inherent to the medical profession not to consider patients from a purely biomedical model but that psychosocial factors should be incorporated into patient care, which forms the foundation of being an advocate [54]. Likewise, we may expect this to be part of the professionalism expectations of every discipline involved in the care process of the patient. However, to date cancer care organization has not been able to find an effective way to enable the patients’ voice to be heard in the MDTMs. As such, more research is needed to have an understanding of how we can change the current ‘medical forum’ culture of current cancer MDTMs into a true patient-centred approach.

Our study has some limitations. We performed a qualitative interview study with non-physician care professionals from three academic hospitals and five non-academic hospitals in one country. Since the Belgian MDTM (MOC) is legally regulated by law, this limits the generalizability of the results for broader organizational contexts and health systems. Additionally, as described above, the multitude of names and competence profiles of nurses working in oncology impede differentiation in the nursing group. In our study, the only differentiation made is between oncological nurses (holding a bachelor degree with additional training in oncology and a care function) and specialist nurses (holding an academic degree and a coordinating function). Further research is needed to discover whether our findings are parallel across oncology departments nationally and internationally. Lastly, this study only focussed on the point of view and experiences of non-physician care professionals. In a previously published study, the same research team conducted a study exploring physicians’ attitudes and perspectives regarding the uptake of psychosocial aspects and/or patient preferences during cancer MDTMs. Both studies can therefore be considered in parallel to get the full picture of what is going on from all points of view of all stakeholders involved in cancer multidisciplinary team meetings.
